# Changes in diagnostic and care trajectories following use of continuous EEG monitoring for neuroprognostication after out of hospital cardiac arrest – a before-and-after study

**DOI:** 10.1016/j.resplu.2026.101268

**Published:** 2026-02-12

**Authors:** Luuk Wieske, Jeroen Hoogland, Ineke van de Pol, Anna J. Court, Vera Lagerburg, Laurien L. Teunissen, Mirjam Datema, Jolande van Helden, Hazra S. Moeniralam, Lea M. Dijksman, Erik Scholten, Antje A. Seeber, Mariska M.G. Leeflang

**Affiliations:** a Department of Clinical Neurophysiology, St. Antonius Hospital, Nieuwegein, the Netherlands; b Department of Neurology and Clinical Neurophysiology, Amsterdam University Medical Centers, Amsterdam, the Netherlands; c Department of Epidemiology and Data Science, Amsterdam Public Health Research Institute, Amsterdam University Medical Centers, Amsterdam, the Netherlands; d Department of Intensive Care, St. Antonius Hospital, Nieuwegein, the Netherlands; e Department of Medical Physics and Instrumentation, St. Antonius Hospital, Nieuwegein, the Netherlands; f Department of Value Based Health Care, St. Antonius Hospital, Nieuwegein, the Netherlands

**Keywords:** Cardiac arrest, EEG, Neurological prognosis, Diagnostic accuracy

## Abstract

**Aim:**

Continuous electro-encephalography (cEEG) has been added to multimodal protocols to improve neurological prognostication in patients after cardiac arrest. Our aim was to investigate the impact of adding cEEG on diagnostic and care trajectories in clinical practice.

**Methods:**

Before-and-after study of patients after out of hospital cardiac arrest admitted in 2013–2024 to the intensive care unit in the Netherlands. Data on diagnostic and care trajectories and costs associated with prognostication were compared between patients admitted from 2013 to 2019, when cEEG was not used to predict the neurological prognosis, and patients admitted from 2019 to 2024, when cEEG was used.

**Results:**

581 patients were included; 355 when cEEG was not used for neuroprognostication and 226 when cEEG was used. cEEG patterns associated with a poor prognosis were found in 18% of patients; cEEG patterns associated with a good prognosis in 24%. When cEEG was used, the neurologic outcome (either good or poor) could be predicted in an additional 23% of patients (95% CI: 16–31%). ICU length of stay did not change when cEEG was used (adjusted estimate: 1% increase in days; 95% CI: −23% to 32%) nor did overall survival (adjusted hazard ratio: 0.93; 95% CI: 0.68–1.27). Overall costs did not differ (estimated difference €1040; 95% CI: −4672 to 7078).

**Conclusion:**

Use of cEEG increased the number of patients in whom the neurological prognosis after out of hospital cardiac arrest could be predicted correctly. We did not observe changes in subsequent care trajectories although this may have been influenced by self-fulfilling prophecies and/or incorporation bias.

## Introduction

In recent years, electro-encephalography (EEG) has been added to (inter)national guidelines as a prognostic modality to determine neurological prognosis in patients after cardiac arrest.^1^, ^2^ For neuroprognostication, EEG patterns are used in addition to other predictors like clinical examination, somato-sensory evoked potentials (SSEP), blood biomarkers and imaging.

Either routine EEG recordings or continuous EEG (cEEG) monitoring may be used. Adding cEEG to multimodal prognostic protocols can increase the sensitivity with which poor outcomes can be predicted and allow good outcomes to be predicted within the first 12 h. However, it is unclear whether cEEG is superior to routine EEG recordings.^3^, ^4^ In these studies, cEEG was used as research tool and data on performance in clinical practice is lacking. Also, it is unknown how cEEG impacts other predictors — particularly predictors that are used after cEEG results are known, such as somatosensory evoked potentials (SSEP). Studies have shown that SSEP can still predict a poor outcome when cEEG patterns are not prognostic.^5^ It is unknown if use and results of SSEP change when cEEG is used first.

Addition of cEEG may also affect care organization and use. A modeling study reported that treatment withdrawal based on cEEG results could lead to a reduction in Intensive Care Unit (ICU) length of stay and reduction of costs but observed data from clinical practice is lacking.^6^

The goal of this study was to investigate if the addition of cEEG to multimodal prognostic outweighs the extra burden that cEEG places on specialized personnel, equipment and expertise. The scarcity of literature addressing our research question highlights the methodological challenges associated with this type of research. The most rigorous prospective design, which would involve study arms using and not using cEEG, is no longer considered ethical. A prospective before-and-after design, would require considerable time and effort starting years before cEEG use. While we acknowledge the methodological pitfalls of retrospective designs, they remain a viable option that can provide valuable insights in challenging research areas. Hereto, we compared diagnostic and care trajectories in a historical cohort of patients after cardiac arrest on the ICU, before and after cEEG was introduced for multimodal neuroprognostication. We hypothesized that the addition of cEEG reduces ICU length of stay due to earlier and more frequent prediction of neurological outcomes.

## Methods

### Design and ethical approval

Historical before-and-after cohort study including patients admitted to the ICU of the St Antonius hospital from January 2013-December 2024. This study period covers two periods: (1) 2013–2019 when cEEG was not used for neuroprognostication and (2) 2019–2024 when cEEG was used for neuroprognostication. The medical ethical committee waived the need for informed consent because only data already collected as part of routine care was used for this study (approval number W22.255). Patients who (had) objected to the use of their hospital data for scientific research were excluded.

### Screening and inclusion

Hospital and ICU databases were examined to identify ICU admissions due to cardiac arrest. Comatose adult patients were included when admitted after out of hospital cardiac arrest (OHCA) of any cause, treated with targeted temperature management (TTM) and when they survived the first 24 h of ICU admission. Patients were excluded if location of cardiac arrest or the neurological outcome at ICU discharge could not be determined from the patient file.

### Setting, prognostic and treatment protocols during the study period

The St. Antonius hospital is a large, non-academic, teaching hospital specialized in cardiovascular care, with a 28–32 bed ICU. Local prognostic protocol guidelines followed national guidelines, which are based on a sequential, multimodal protocol and is described in detail in the supplementary material. In short, from 2013 to 2019, a neurological examination without sedation was performed first at least 24 h after Return of Spontaneous Circulation (ROSC). In case a motor score <3 was found and bilateral absent pupillary responses or bilaterally absent SSEP (N20) responses the neurological prognosis was poor. In 2019, following an adaption of national guidelines, cEEG was added to the multimodal prognostic protocol. cEEG patterns at 12 and 24 h after ROSC were retrospectively interpreted in case a neurological assessment at least 24 h after ROSC performed without sedation showed a motor score <3. The following cEEG patterns are associated with a poor prognosis: (1) *iso*-electric or suppressed (<10 µV) EEG from 12 h after ROSC; (2) low voltage (<20 µV) EEG from 24 h; (3) burst suppression with identical bursts from 24 h; and (4) generalized periodic discharges (GPDs) on an *iso*-electric background from 24 h. A continuous background pattern within 12 h after ROSC was associated with a good prognosis but this information did not change care (like earlier cessation of sedation).^3^ Interpretation of cEEG and SSEP was done by clinical neurophysiologists as part of routine care and not blinded. Use of routine EEG, imaging and blood biomarkers is described in the supplement.

When a neurological prognosis was established – based on the results of the neurological examination, cEEG, and/or SSEP – it was discussed within the clinical team and with family members. The goal was to reach consensus on whether to continue or withdraw treatment, while also considering the patient’s previously expressed wishes. The clinical team was not restricted to follow multimodal protocol predictions.

During the study period, ICU treatment of patients after OHCA remained unchanged, using propofol and remifentanil sedation and targeted temperature management (TTM). The target temperature was changed from 32–34°C to 36°C in 2015. In the 2013–2019 period, cEEG was available for use in ICU patients but only to monitor for seizures and not for neuroprognostication.

### Data extraction

Data were extracted into an electronic case record form from the electronic patient file and neurophysiology databases by the investigators. Only data for the first OHCA and the first ICU admission following that OHCA were extracted. Data on patient demographics, comorbidities, and cardiac arrest characteristics were extracted. ICU admission data included length of stay, (presumed) seizures, discharge location and the primary cause of death in case of death on the ICU. Data on pupillary responses was collected and was scored as absent or as not (reliably) absent in case pupillary responses were either present, could not be tested without sedation or when other factors, like ophthalmologic conditions affecting pupillary responses could not be ruled out. At ICU discharge, hospital discharge, and at 3, 6 and 12 months after cardiac arrest, the cerebral performance score (CPC) was retrospectively assessed by the investigators based on chart review. The neurological prognosis was dichotomized into ‘good’ (CPC score 1–2) and ‘poor’ (CPC 3–5). CPC 3 due to a temporary delirium during ICU discharge was scored as CPC 2. In case of insufficient detail in charts at a specific time point, or in case a new neurologic event impacting the CPC occurred during follow-up, the last CPC score before that time point was used (last observation carried forward). Survival up to 12 months after cardiac arrest was extracted from the electronic patient file or the last date confirmed alive was recorded, in case patients were lost to follow-up.

From the neurophysiological databases the start and stop times and interpretation of cEEG and SSEP were extracted based on the original reports. In case cEEG patterns at 12 or 24 h following ROSC were not detailed in the original report, these were retrospectively assessed by the investigators, which were not blinded to study information.

### Outcomes

To investigate changes in diagnostic trajectories, the following outcomes were analyzed: (1) the difference in the number of patients in whom the neurological prognosis was predicted by the multimodal prognostic protocol, (2) utilization and results of SSEP, pupillary responses and overlap with cEEG patterns, and (3) false positive cEEG patterns predicting a poor outcome.

To investigate changes in care trajectories: (1) differences in ICU length of stay with or without cEEG for neuroprognostication, (2) differences in survival up to 12 months and, (3) differences in costs associated with neuroprognostication.

### Statistical analysis

Differences between categorical variables were assessed using a Chi square or Fisher’s exact test, differences between continuous variables using a *t*-test or a Mann-Whitney *U* test depending on the distribution. An alpha level of 5% was used throughout. Where appropriate, proportions and differences between proportions are shown with corresponding 95% confidence intervals (95% CI).

Differences in ICU length of stay were analyzed using a negative binomial regression model to accommodate overdispersed count data.^7^ An interrupted time series design was used to account for changes over time in ICU length of stay and was based on the hypothesis that introduction of cEEG might have led to a level change.^8^ Two time periods were used, before and after adaptation of the national guideline. Effects were estimated unadjusted and adjusted for (potential) length of stay confounders (age, gender, APACHE IV score, cardiac or non-cardiac arrest, (presumed) seizures (coded as yes/no)).^9^ Missing APACHE IV scores (missing because of registration problems) were imputed before regression analyses using multiple imputation with chained equations. The full dataset was used for imputation and estimates of regression coefficients were pooled across 10 imputed datasets.

Differences in survival were analyzed using a Kaplan-Meier curve and a Cox-regression model, pooling estimates across 10 imputed datasets. Hazard ratio’s (HR) were estimated unadjusted and adjusted for (potential) mortality confounders (age, gender, APACHE IV score, cardiac or non-cardiac arrest, (presumed) seizures (coded as yes/no)). An overview was created for costs associated with neuroprognostication and costs were compared between the two time periods (details are described in the supplementary material).

There is no previous data available to support a sample size calculation and all eligible patients within the time period were included. As a sensitivity analysis, we performed a per-protocol analysis focusing on patients in whom the prognostic multimodal protocol with or without cEEG was followed as intended (see supplement).

Analyses were performed in R (version 4.4.0).

## Results

**Fig. 1:**
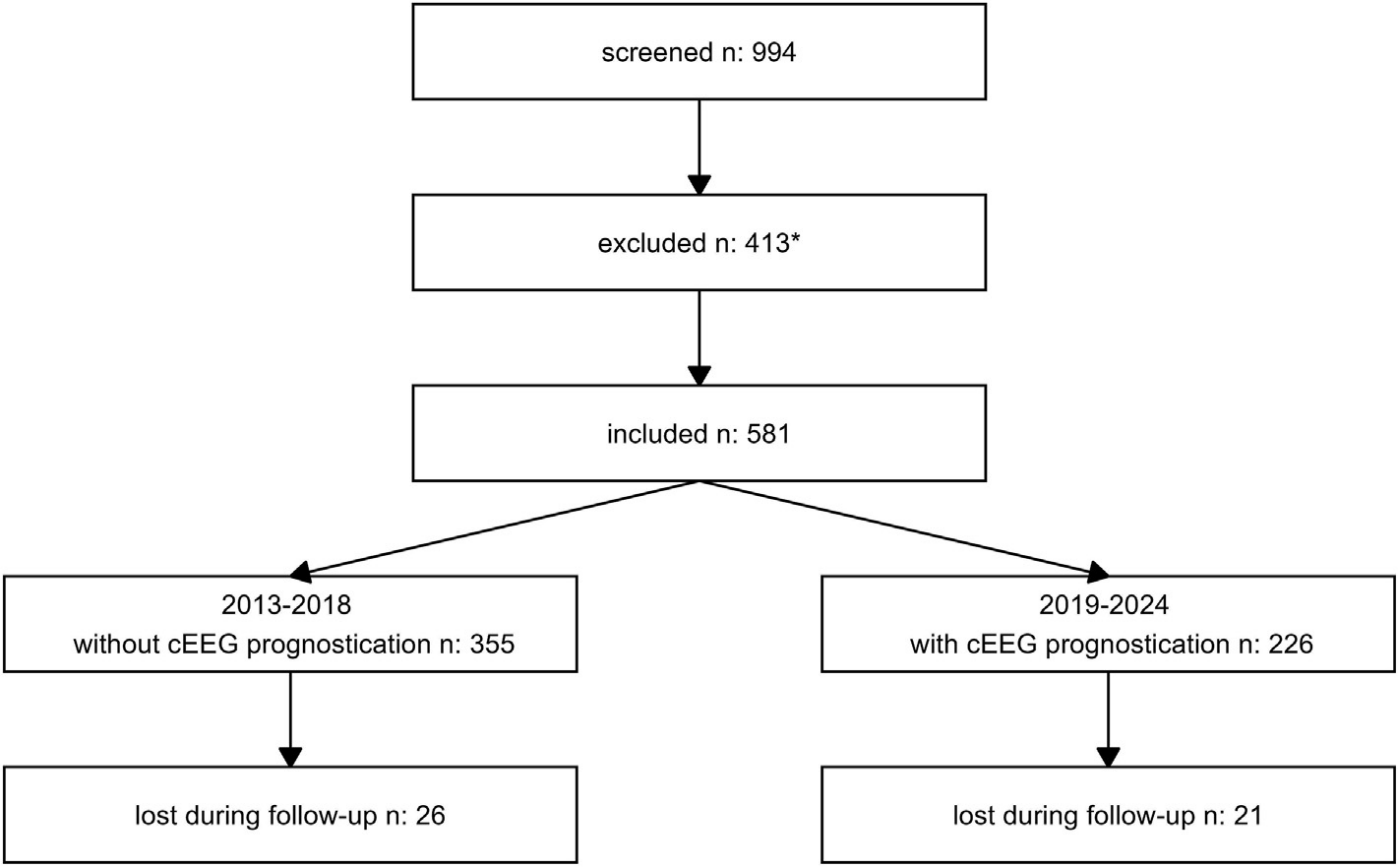
**Study flowchart**. *Reasons for exclusion: 1 no consent, 44 missing data, 27 referral other hospital, 39 double records, 234 no out of hospital cardiac arrest, 59 length of stay on the ICU < 24 h, 9 no targeted temperature management. cEEG: continuous EEG.

We included 581 patients; 355 when cEEG was not used for neuroprognostication and 226 when cEEG was used (Fig. 1). Table 1 shows patient characteristics. During the period cEEG was used for neuroprognostication, non-cardiac reasons for the arrest tended to occur more frequently and patients had higher APACHE IV scores.

**Table 1:** Cohort characteristics. Table showing cohort characteristics for patients after cardiac arrest between 2013 and 2019 when continuous EEG (cEEG) monitoring was not used for neuroprognostication and 2019–2024 when cEEG was used for neuroprognostication.

		**Without cEEG prognostication** **(*N*: 355)**	**With cEEG prognostication** **(*N*: 226)**	***p*-value**
Age in years		64 ± 13	64 ± 15	0.82
Male sex		269 (75.8%)	164 (72.6%)	0.44
Presumed cause of cardiac arrest				0.06
	Cardiac	283 (79.7%)	161 (71.2%)	
	Non-cardiac	63 (17.7%)	54 (23.9%)	
	Unknown	9 (2.5%)	11 (4.9%)	
First initial rhythm*				0.26
	Shockable	242 (71.0%)	144 (66.1%)	
	Non-shockable	99 (29.0%)	74 (33.9%)	
Estimated duration of resuscitation in minutes		16 (10–30)	17 (10–24)	0.95
APACHE IV score°		105 (80–128)	115 (101–133)	<0.01

All variables are in number (%), mean (± SD) or median (25–75%).

cEEG: continuous EEG; APACHE IV: Acute Physiology and Chronic Health Evaluation IV.

* Data missing in 22 patients.

° Data missing in 122 patients.

### Diagnostic trajectories before and after use of cEEG for prognostication

**Fig. 2:**
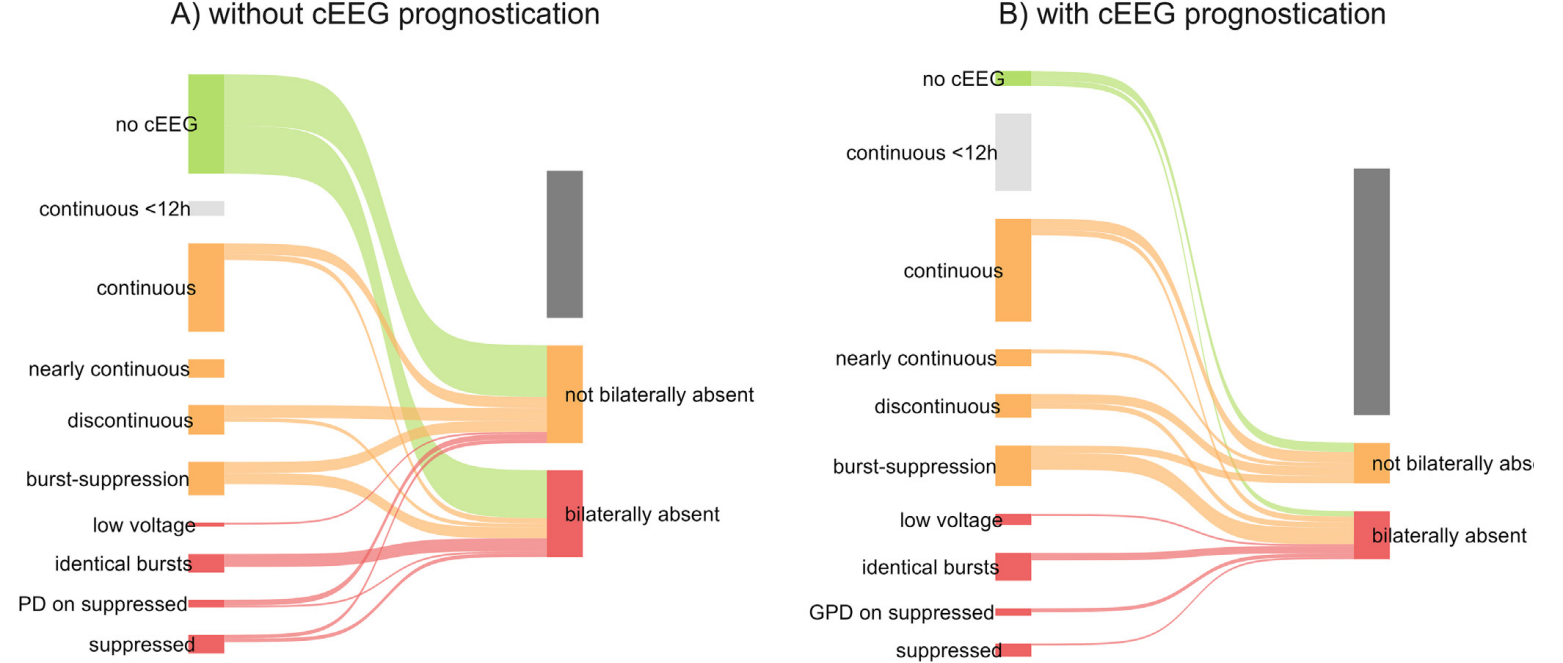
**Overlap between continuous EEG (CEEG) patterns and somato-sensory evoked potentials (SSEP) results**. Sankey plot showing the overlap between continuous EEG (cEEG) patterns and somato-sensory evoked potentials (SSEP) results in patients in whom at least one of these tests were made for the time period when cEEG was not used for prognostication (A) and for the time period when cEEG was used for prognostication (B). cEEG or SSEP results associated with a poor prognosis are shown in red, with an unclear prognosis in orange and with a good prognosis is green. cEEG: continuous EEG; GPD: generalized periodic discharge; SSEP: somato-sensory evoked potentials. (For interpretation of the references to color in this figure legend, the reader is referred to the web version of this article.)

Table 2 describes results and prognostic value of pupillary responses, SSEP and cEEG before and after cEEG was used. Supplementary Table 1 provides additional results and other outcome predictors. After cEEG use, a SSEP was ordered in 21.2% (48/226) patients, which was less compared to before cEEG use (difference in proportions: −6.9%; 95% CI: 0 to −14%). Fig. 2 shows overlap between cEEG patterns and SSEP responses in patients in whom at least one of these tests was performed. In case of an identical burst suppression pattern on cEEG, SSEP (N20) responses were bilaterally absent in all recorded patients. Other cEEG patterns were not uniformly associated with SSEP (N20) responses.

**Table 2:** Results and prognostic value of outcome predictors. Table showing results for prognostic modalities predicting neurological outcome after cardiac arrest.

		**Without cEEG prognostication**	**With cEEG prognostication**	***p*-value**
PR bilaterally absent		34/355 (9.6%)	28/226 (12.4%)	0.35
SSEP performed		100/355 (28.2%)	48/226 (21.2%)	0.08
SSEP bilaterally absent		47/100 (47%)	26/48 (54.2%)	0.52
cEEG performed		136/355 (38.3%)	184/226 (81.4%)	n.a.
Prognosis associated with cEEG pattern	Good*	8/136 (6.3%)	42/184 (24.0%)	<0.01
	Poor	26/136 (20.5%)	32/184 (18.3%)	
	Unclear	93/136 (73.2%)	101/184 (57.7%)	
**Prediction of good CPC score at 12 months**				
Supported by cEEG		n.a.	37/111 (33.3%)*	n.a.
**Prediction of poor CPC score at 12 months**				
Supported by PR only		24/174 (13.8%)	11/115 (9.6%)	n.a.
Supported by SSEP only		37/174 (21.3%)	16/115 (13.9%)	
Supported by SSEP and PR		10/174 (5.7%)	2/115 (1.7%)	
Supported by cEEG only		n.a.	11/115 (9.6%)	
Supported by cEEG and PR			13/115 (11.3%)	
Supported by cEEG and SSEP			6/115 (5.2%)	
Supported by cEEG, SSEP and PR			2/115 (1.7%)	
Supported by either PR, SSEP or cEEG		71/174 (40.8%)	61/115 (53.0%)	0.05

All variables are in number (%) or median (25–75%).

cEEG: continuous EEG; SSEP: somatosensory evoked potential; ROSC: return of spontaneous circulation; PR: pupillary response; n.a.: not applicable.

* Of the in total 50 patients with a cEEG pattern associated with a good neurological prognosis 6 died (4 in the ICU (1 because of neurological reasons), 1 in hospital and 1 patient after hospital discharge).

The number of patients in whom the neurological prognosis (either good or poor) was predicted correctly by the multimodal protocol increased from 20% (71/355), when cEEG was not used for neuroprognostication, to 43.3% when cEEG was used (98/226; difference in proportions: 23%; 95% CI: 16–31%). Table 2 shows these changes separately for patients with a good or poor outcome at 12 months.

There were no cases of a poor cEEG pattern in patients with a good neurological outcome at 12 months either in the period when cEEG was not used (0/127 patients in whom a cEEG was started within 24 h after cardiac arrest; 95% CI: 0–3.7%) or in the period when cEEG was used (0/175 patients in whom a cEEG was started within 24 h after cardiac arrest; 95% CI: 0–2.7%).

### Care trajectories before and after use of cEEG for prognostication

Overall ICU length of stay was 4 days (IQR: 3–7) and did not differ between the time periods (*p*: 0.24; Table 3). Supplementary Fig. 1 shows the trend over time in ICU length of stay during the study period. Use of cEEG for neuroprognostication was not associated with a change in ICU length of stay (unadjusted estimate of 3% increase in days admitted (95% CI: −21% to 34%) and 1% increase in days (95% CI −23% to 32%) adjusted for confounders).

**Table 3:** Outcomes after cardiac arrest. Table showing outcomes during ICU admission, hospital admission and follow-up up to one year after cardiac arrest.

		**Without cEEG prognostication** **(*N*: 355)**	**With cEEG prognostication** **(*N*: 226)**	***p*-value**
ICU LOS in days		4 (3–7)	4 (3–7)	0.24
ICU mortality		152/355 (42.8%)	99/226 (43.8%)	0.94
	Treatment withdrawal mainly because of poor neurological prognosis	95/152 (62.5%)	67/99 (67.7%)	0.96
	Treatment withdrawal because of poor overall prognosis	20/152 (13.2%)	13/99 (13.1%)	
	Brain death	9/152 (5.9%)	4/99 (4.0%)	
	Cardiogenic shock	12/152 (7.9%)	9/99 (9.1%)	
	Multi-organ failure	5/152 (3.3%)	2/99 (2.0%)	
	Sepsis	5/152 (3.3%)	2/99 (2.0%)	
	Other	4/152 (2.6%)	2/99 (2.0%)	
	Unknown	2/152 (1.3%)	0/99 (0%)	
**CPC score at ICU discharge**				
	Good	188/355 (53.0%)	114/226 (50.4%)	0.61
	Poor	167/355 (47.0%)	112/226 (49.6%)	
Hospital LOS in days		17 (11–27)	16 (11–26)	0.87
In hospital mortality		9/355 (2.5%)	8/226 (3.5%)	0.65
**Discharge location from hospital**				
	Home	144/194 (74.2%)	87/119 (73.1%)	0.74
	Rehabilitation center	30/194 (15.5%)	23/119 (19.3%)	
	Nursing home	8/194 (4.1%)	4/119 (3.4%)	
	Other	12/194 (6.2%)	5/119 (4.2%)	
Mortality during follow-up*		169/355 (47.6%)	114/226 (50.4%)	0.56
**CPC score at 12 months**				
	Good	181/355 (51.0%)	111/226 (49.1%)	0.73
	Poor	174/355 (49.0%)	115/226 (50.9%)	

All variables are in number (%) or median (25–75%).

cEEG: continuous EEG; SSEP: somatosensory evoked potential; ICU: Intensive Care Unit; LOS: length of stay; CPC: cerebral performance category.

* 47 patients were censored during follow-up (see flowchart).

**Fig. 3:**
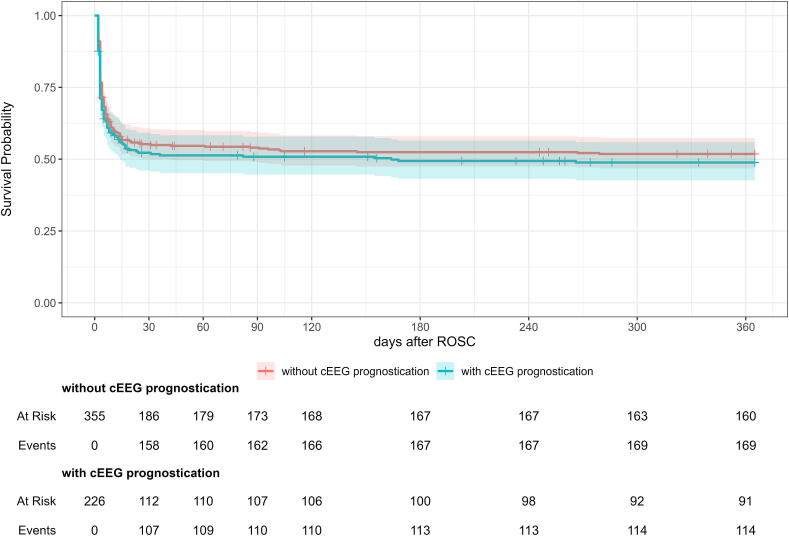
**Survival curve**. Kaplan-Meier curve showing survival up to one year after cardiac arrest. Patients in whom continuous EEG monitoring (cEEG) was not used for neuroprognostication are shown in red; patients in whom cEEG was used in blue. cEEG: continuous EEG; ROSC: return of spontaneous circulation. (For interpretation of the references to color in this figure legend, the reader is referred to the web version of this article.)

At 12 months after cardiac arrest, 50.3% (292/581) of all patients had a good neurological outcome and this did not differ between the time periods (Table 3). Fig. 3 shows the survival curve. Use of cEEG for neuroprognostication was not associated with survival at 12 months (unadjusted estimated HR 1.11 (95% CI: 0.88–1.41) and 0.93 (95% CI 0.68–1.27) adjusted for confounders).

Supplementary Table 2 shows costs associated with neuroprognostication. There was a potentially small reduction in costs associated with SSEP and increase in cEEG costs when cEEG was used for neuroprognostication. Costs for the number of ICU or hospital days and total costs did not differ between the two time periods.

### Sensitivity analysis

Results of the per-protocol analysis are shown in the supplement. In the per-protocol population, use of cEEG was associated with a reduction in ICU length of stay and not with survival at 12 months.

## Discussion

Use of cEEG increased the number of patients in whom the neurological prognosis after out of hospital cardiac arrest could be predicted. cEEG predicted a good prognosis correctly in 33.3% of patients with a good outcome – which is not possible with other predictors – and an additional 12.2% of patients with a poor prognosis were identified when cEEG was used. Impact on the utilization and results of other predictors was minimal or absent. Overall, introduction of cEEG did not result in changes to care trajectories or associated costs.

cEEG enabled earlier prediction of a good prognosis. Although a good prognosis is also evident in most patients shortly after sedation is stopped, earlier prediction using cEEG may have some clinical implications as it helps to justify aggressive treatment of other problems, like hemodynamic failure.

For poor outcome, we observed an increase of 12.2% additional patients identified when cEEG was used. Although this is highly relevant for individual patients, on a group level this change is modest. This may be explained because cEEG patterns associated with a poor prognosis were observed in only 18.3% of patients, similar to other studies, and the majority of cEEG patterns observed had an unclear association with prognosis.^3^, ^4^ Also, we observed that cEEG patterns associated with a poor outcome frequently overlapped with other predictors indicating a poor outcome.

In general, increases in sensitivity for a diagnostic instrument are paralleled by a decreases in specificity.^10^ The increased sensitivity in predicting a poor outcome when using cEEG might therefore theoretically decrease specificity. False positive prediction of a poor neurological outcome is a critically relevant issue because of the self-fulfilling prophecy problem.^11^ We did not observe false positive cEEG predictions of a poor outcome but we did not investigate the influence of the self-fulfilling prophecy on this estimate. Survival after cardiac arrest may be seen as a crude estimate of false positives; we did not observe changes in survival when cEEG was used.

The currently limited clinical relevance of early good outcome prediction and modest impact of cEEG on poor outcome prediction may explain why we did not observe changes in ICU length of stay or costs associated with neuroprognostication. Using a per-protocol analysis, we did observe a reduction in ICU length of stay with cEEG, indicating that protocol non-adherence may also be explanation. We did not study reasons for non-adherence. Because we focused on care organization and use as endpoints, we chose ICU length of stay and costs. It should be noted these endpoints are not only influenced by establishing an reliable (and early) prognosis but also by other factors, ranging from concomitant problems, like cardiogenic shock, to discharge policies.

This study has several limitations. First, we chose a before-and-after design using a historical cohort as this design provided the best tradeoff between (ethical) feasibility and the level of detail needed to still allow valid results. A prospective design could however have allowed for more detailed data, like a day-to-day evaluation of the probability of good or poor outcome, or a time-to-definitive prognosis estimate. Not all the different tests were performed in each patient which may have led to sampling bias. Our results are not generalizable to parallel multimodal protocols. We did not compare cEEG findings to imaging or biomarker results because these were not routinely used. To account for changes throughout the study period, we have used an interrupted time series design for the main analyses that was adjusted for key potential confounders but we could not correct for all potential confounders (like changes in withdrawal of care policies) and therefore cannot rule out residual confounding. Because of extended study period, standardized EEG scoring guidelines, like the 2021 ACNS guidelines, were not implemented.^12^ A relatively large number of patients during the 2015–2019 period were monitored using cEEG because of participation in the TELSTAR trial.^13^ Although these cEEGs were only used to monitor for seizures, we cannot exclude potential incorporation bias of cEEG patterns, which may have underestimated the number of false positive results. In addition, potential incorporation bias may have reduced effects of cEEG on ICU length of stay, as indicated by our per-protocol analysis. Costs analyses were restricted to an overview of costs directly associated with neuroprognostication while costs or benefits of multimodal protocols involving cEEG may extend beyond these factors. This study was performed without formal sample size estimation and may have been underpowered.

The impact of cEEG on diagnostic and care trajectories may be improved by new cEEG patterns associated with a poor or good prognosis, especially patterns that are independent of other predictors like SSEP or pupillary responses. Artificial intelligence based EEG interpretation may improve prognostic accuracy.^14^ Also, studies are currently exploring if standard treatment protocols involving 24 h sedation can be shortened in patients with a good neurological prognosis based on cEEG prediction.^15^

## Conclusion

Use of cEEG increased the number of patients in whom the neurological prognosis after out of hospital cardiac arrest could be predicted correctly. We did not observe changes in subsequent care trajectories although this may have been influenced by self-fulfilling prophecies and/or incorporation bias.

## CRediT authorship contribution statement

**Luuk Wieske:** Writing – review & editing, Writing – original draft, Methodology, Investigation, Formal analysis, Conceptualization. **Jeroen Hoogland:** Writing – review & editing, Writing – original draft, Methodology. **Ineke van de Pol:** Writing – review & editing, Writing – original draft, Methodology. **Anna J. Court:** Writing – review & editing, Writing – original draft. **Vera Lagerburg:** Writing – review & editing, Writing – original draft, Methodology. **Laurien L. Teunissen:** Writing – review & editing, Writing – original draft. **Mirjam Datema:** Writing – review & editing, Writing – original draft. **Jolande van Helden:** Writing – review & editing, Writing – original draft. **Hazra S. Moeniralam:** Writing – review & editing, Writing – original draft. **Lea M. Dijksman:** Writing – review & editing, Writing – original draft, Formal analysis. **Erik Scholten:** Writing – review & editing, Writing – original draft. **Antje A. Seeber:** Writing – review & editing, Writing – original draft, Conceptualization. **Mariska M.G. Leeflang:** Writing – review & editing, Writing – original draft, Supervision.

## Funding

This research did not receive any specific grant from funding agencies in the public, commercial, or not-for-profit sectors.

## Declaration of competing interest

None.
