# Supplementary material for: Changes in diagnostic and care trajectories following use of continuous EEG monitoring for neuroprognostication after out of hospital cardiac arrest – a before-and-after study
Source: Resusc Plus. 2026 Feb 12;28:101268. doi: 10.1016/j.resplu.2026.101268 (PMC13080491; doi:10.1016/j.resplu.2026.101268)
Supplement: Supplementary Data 1 [file mmc1.pdf]

Changes in diagnostic and care trajectories following use of continuous EEG monitoring for neuroprognostication after out of hospital cardiac arrest – a before-and-after study

Luuk Wieske, Jeroen Hoogland, Ineke van de Pol, Anna Court, Vera Lagerburg, Laurien L. Teunissen, Mirjam Datema, Jolande van Helden, Hazra S. Moeniralam, Lea M. Dijksman, Erik Scholten, Antje A. Seeber, Mariska M.G. Leeflang

Supplementary material

Contents

Multimodal prognostic protocol – pag 2 & 3

Cost overview analysis – pag 3

Supplementary Figure 1: ICU length of stay over time – pag 4

Supplementary Table 1: Detailed results and prognostic value of outcome predictors – pag 5

Supplementary Table 2: Cost overview of neuroprognostication – pag 6

Supplementary analysis: per protocol sensitivity analysis – pag 7

References – pag 8

Multimodal prognostic protocol

Throughout the study period, neuroprognostication was performed as part of routine care by a multidisciplinary team of intensivists, neurologists and clinical neurophysiologists. Local prognostic protocol guidelines followed national guidelines which are based on a sequential, multimodal approach to outcome prediction.[1] From 2013 to 2019, neuroprognostication began with a clinical examination, at least 24 hours after Return of Spontaneous Circulation (ROSC), after sedation was stopped and no longer affected the neurological assessment. Patients with bilateral absent pupillary responses were judged to have a poor prognosis and, in principle, no additional tests were performed. In patients with intact pupillary responses and a motor score <3, a SSEP was performed. In case a median or ulnar nerve SSEP showed a bilaterally absent N20 response with retained N9 and N13 responses, the neurological prognosis was poor. In case the N20 response was not bilaterally absent, the neurological prognosis at that moment was unclear and patients were followed over time by the clinical team.

From 2019, following an adaptation of the national guidelines (dated on 15-2-2019[1]), cEEG patterns were added into the multimodal prognostic protocol. cEEG was started as soon as possible after ICU admission, preferably within 24 hours after ROSC. In case a neurological assessment at least 24 hours after ROSC and performed without sedation showed a motor score <3, cEEG patterns at 12 and 24 hours after ROSC were retrospectively interpreted. The following cEEG patterns are associated with a poor prognosis: 1) iso-electric or suppression (<10 microvolt) EEG from 12 hours after ROSC; 2) low voltage (<20 microvolt) EEG from 24 hours; 3) burst suppressions with identical bursts from 24 hours; and 4) generalized periodic discharges (GPDs) on an iso-electric background from 24 hours. A continuous background within 12 hours after ROSC was associated with a good prognosis. The prognostic association of cEEG patterns not fulfilling these criteria was classified as unclear. SSEP was used as an additional test after cEEG and could be performed when cEEG patterns were non-prognostic or when the clinical team wanted more certainty.

In addition to pupillary responses, SSEP and cEEG, national and local guidelines permitted to use of routine EEG recordings, neuroimaging and blood biomarkers (neuron-specific enolase; NSE) to predict the neurological prognosis. Routine EEG recordings could be used around 72 hours after ROSC and a suppressed or low voltage (<20 microvolt) background pattern was associated with a poor prognosis. Either CT or MRI could also be used to provide

prognostic information and was usually used in later stages (after several days to one week) in case the prognosis was still unclear. Diffuse edema on CT was associated with a poor prognosis and other imaging abnormalities were not unequivocally associated with a poor prognosis. NSE levels >65 µg/L at 48 and 72 hours were also associated with a poor prognosis but were not used in isolation. Data on routine EEG recordings and neuroimaging is shown in supplementary table 1. Data on NSE is not reported as this was only implemented in 2023 making comparison between the time period before and after cEEG impossible.

Cost overview analysis

A cost overview was created for costs associated with neuroprognostication. This was defined as costs for each day of ICU, each day of hospital admission and costs for SSEP and/or cEEG. We used the following reference prices based on the Dutch National Health Institute (2024): one day in the ICU €2722, one day in the hospital €644.[2] Costs for other procedures were not included in this database and for these procedures we created estimates that were based on nonvariable costs (costs for purchasing equipment and maintenance) and variable costs (for using equipment and hourly fees for EEG technicians and clinical neurophysiologists). This resulted in the following estimates of €400 for SSEP, €332 for starting a cEEG registration and €203 for each additional day of cEEG monitoring. Mean costs and differences in means between the two time periods were calculated using bias-corrected and accelerated (BCA) bootstrapping (N:5000) and are shown with the 95% confidence interval.[3]

Supplementary Figure 1: ICU length of stay over time

Figure showing trend over time during the study period for length of stay in the Intensive Care Unit (ICU) in days following out of hospital cardiac arrest. The blue line is a smoothed regression line to illustrate the trend over time. The period when continuous EEG (cEEG) was used for neuroprognostication is marked by the grey time period.

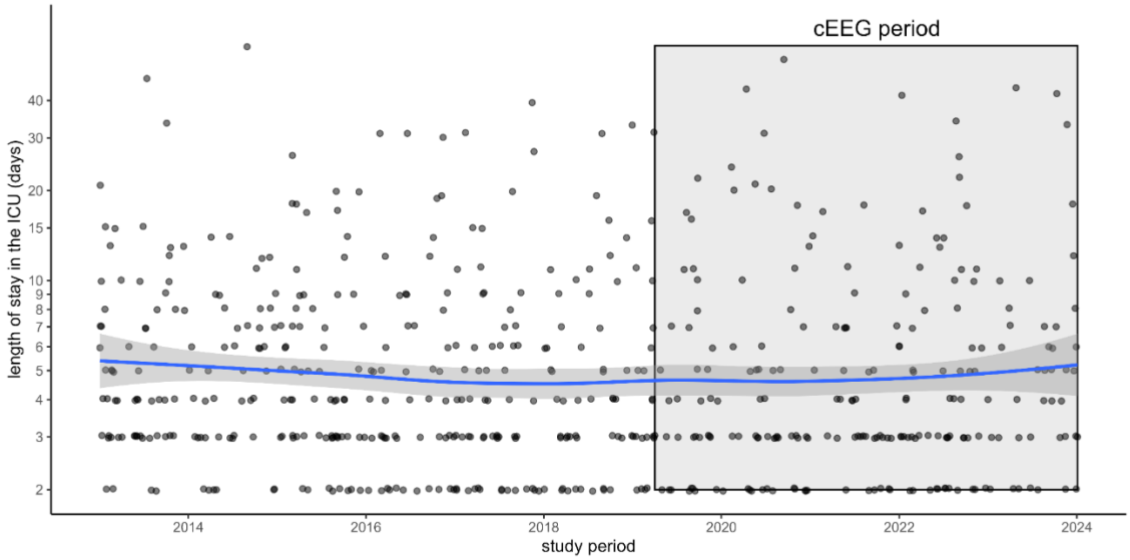

ICU: Intensive Care unit

Supplementary Table 1: Detailed results and prognostic value of outcome predictors

Table showing additional results for prognostic modalities predicting neurological outcome after cardiac arrest.

	without cEEG prognostication	with cEEG prognostication
SSEP performed	100/355 (28.2%)	48/226 (21.2%)
day of SSEP after ROSC	2 (2-3)	2 (2-3)
cEEG performed	136/355 (38.3%)	184/226 (81.4%)
start time cEEG after ROSC in hours	16 (9-20)	11 (6-17)
cEEG running at T=12 after ROSC	45/136 (33.1%)	97/184 (52.7%)
cEEG running at T=24 after ROSC	127/136 (93.4%)	175/184 (95.1%)
cEEG duration in hours	28 (23-49)	28 (23-44)
rEEG performed	45/355 (12.7%)	14/226 (6.2%)
rEEG indicative of a poor prognosis	16/45 (36.0%)	3/14 (21.4%)
CT performed for prognosis	5/355 (1.4%)	5/226 (2.2%)
MRI performed for prognosis	1/355 (0.3%)	10/226 (4.4%)

All variabeles are in number (%) or median (25%-75%).

SSEP: somatosensory evoked potential; cEEG: continuous EEG; rEEG: routine EEG recording; ROSC: return of spontaneous circulation

Supplementary Table 2: Cost overview of neuroprognostication

Table showing a cost overview of costs associated with neuroprognostication, i.e. costs for each day of ICU admission, hospital admission and for somatosensory evoked potential (SSEP) and continuous EEG (cEEG). Bootstrapped estimates for the mean for each timeperiod and the difference in means are shown with associated 95% confidence interval (CI)

	without cEEG prognostication (N: 355)	with cEEG prognostication (N:226)	difference in means (95% CI)
Costs ICU LOS, mean (95% CI)	€18057 (16026 to 19943)	€18597 (15573 to 21366)	€539 (-2932 to 4231)
Costs hospital LOS, mean (95% CI)	€7833 (6716 to 8871)	€7754 (4523 to 11036)	€-80 (-3481 to 3332)
Costs SSEP, mean (95% CI)	€113 (94 to 131)	€85 (62 to 104)	€-28 (-56 to 1)
Costs cEEG, mean (95% CI)	€0 (0 to 0)	€443 (387 to 490)	€443 (404 to 514)
Total costs, mean (95% CI)	€26003 (23186 to 28601)	€27043 (21914 to 31911)	€1040 (-4672 to 7078)

cEEG: continuous EEG; SSEP: somatosensory evoked potential; ICU: Intensive Care Unit; LOS: length of stay; CI: confidence interval

Supplementary analysis: per protocol sensitivity analysis

A per-protocol sensitivity analysis was performed according to investigate the effects of cEEG implementation on ICU length of stay and 12-month survival in patients for whom the prognostic multimodal protocol, with or without cEEG, was followed as intended. Although cEEG before 2019 was only used for seizure monitoring and not for prognostication, we also excluded patients who were monitored with cEEG before 2019 for seizures in an effort to minimize incorporation bias. Hereto we created two subgroups: 1) patients (N: 228) admitted before 2019 who were *not* monitored before 24 hours after return of spontaneous circulation using cEEG for seizures and 2) patients (N: 175) admitted in or after 2019 who were monitored with cEEG before 24 hours after return of spontaneous circulation. The proportion of patients with a poor outcome at 12 months did not differ between these two subgroups (102/228 vs 91/175; p: 0.19). Overall ICU length of stay was 4 days (IQR 3-8) for patients without cEEG and 4 days (3-7) for patients with cEEG. In table below we report unadjusted and adjusted interrupted time series negative binomial regression models and cox regression models, recreated in the per-protocol group, as described in the methods section.

	unadjusted	adjusted
Change in ICU length of stay in days when cEEG was used	-33 % (95%: -54 to -0.2)	-39 % (95%: -59 to -0.8)*
Change in survival at 12 months when cEEG was used	HR: 1.3 (95%: 1.0-1.8)	HR: 1.1 (95%: 0.8-1.6)†

*: adjusted for age, gender, APACHE IV score, cardiac or non-cardiac arrest, (presumed) seizures (coded as yes/no)

†: adjusted for age, gender, APACHE IV score, cardiac or non-cardiac arrest, (presumed) seizures (coded as yes/no)

References

1. Prognosis of postanoxic coma. Dutch guideline for healthcare providers, 2019. (Accessed 21 October 2025, at https://richtlijnendatabase.nl/richtlijn/prognose_van_postanoxisch_coma/startpagina.html)

2. National Health Care Institute (NZa). Costing manual: Methods and Reference Prices for Economic Evaluations in Healthcare. National Health Care Institute, 2024 (Accessed 21 October 2025, at https://english.zorginstituutnederland.nl/site/binaries/site-content/collections/documents/2024/01/16/guideline-for-economic-evaluations-in-healthcare/Module+-+Costing+manual.pdf)

3. Barber JA, Thompson SG. Analysis of cost data in randomized trials: an application of the non-parametric bootstrap. Stat Med 2000;19:3219–3236.
